# Advances in Understanding the Human Urinary Microbiome and Its Potential Role in Urinary Tract Infection

**DOI:** 10.1128/mBio.00218-20

**Published:** 2020-04-28

**Authors:** Michael L. Neugent, Neha V. Hulyalkar, Vivian H. Nguyen, Philippe E. Zimmern, Nicole J. De Nisco

**Affiliations:** a Department of Biological Sciences, University of Texas at Dallas, Richardson, Texas, USA; b Department of Urology, University of Texas Southwestern Medical Center, Dallas, Texas, USA; University of Texas Health Science Center at Houston

**Keywords:** metagenomics, microbial communities, microbiome, probiotics, urinary tract infection

## Abstract

Recent advances in the analysis of microbial communities colonizing the human body have identified a resident microbial community in the human urinary tract (UT). Compared to many other microbial niches, the human UT harbors a relatively low biomass. Studies have identified many genera and species that may constitute a core urinary microbiome. However, the contribution of the UT microbiome to urinary tract infection (UTI) and recurrent UTI (rUTI) pathobiology is not yet clearly understood. Evidence suggests that commensal species within the UT and urogenital tract (UGT) microbiomes, such as Lactobacillus crispatus, may act to protect against colonization with uropathogens. However, the mechanisms and fundamental biology of the urinary microbiome-host relationship are not understood. The ability to measure and characterize the urinary microbiome has been enabled through the development of next-generation sequencing and bioinformatic platforms that allow for the unbiased detection of resident microbial DNA. Translating technological advances into clinical insight will require further study of the microbial and genomic ecology of the urinary microbiome in both health and disease. Future diagnostic, prognostic, and therapeutic options for the management of UTI may soon incorporate efforts to measure, restore, and/or preserve the native, healthy ecology of the urinary microbiomes.

## Abstract (precis)

Recent advances in the analysis of microbial communities colonizing the human body have identified a resident microbial community in the human urinary tract (UT). Compared to many other microbial niches, the human UT harbors a relatively low biomass. Studies have identified many genera and species that may constitute a core urinary microbiome. However, the contribution of the UT microbiome to urinary tract infection (UTI) and recurrent UTI (rUTI) pathobiology is not yet clearly understood. Evidence suggests that commensal species within the UT and urogenital tract (UGT) microbiomes, such as Lactobacillus crispatus, may act to protect against colonization with uropathogens.

## INTRODUCTION

The human microbiome is the sum of all genomic information belonging to the resident microbiota, the nonhuman life that colonizes the body. Through years of research, it has become clear that the human body harbors distinct microbial populations within various anatomical niches (1–3). A long understudied but important microbial niche is now being closely examined and functionally characterized. The human urinary tract harbors a resident microbial community (4–8). These relatively new data characterizing the human urinary microbiota defy decades-old theory and clinical practice of urinary tract sterility in urogenital health (5, 9). For the purposes of this review, the term “urinary tract” (UT) refers to the organ system involved in the production, transport, storage, and excretion of urine, namely, the kidney, ureter, bladder, and urethra. Discussion of a larger system, which includes the UT, is also relevant for the purposes of this review. Hence, the term “urogenital tract” (UGT) is used to refer to an organ system which includes the UT as well as the anatomical sites and organs involved in reproduction that may contribute to the urine microbial load, such as the vagina, cervix, periurethral skin, penis, pubic skin surfaces, and perineal area. Depending on the sampling method, the urinary microbiota may consist of species residing within the bladder, UT, or UGT. The urinary microbiota has been characterized and reproducibly measured. To date, >100 species from more than 50 genera are thought to reside in the human UT and UGT (4–6, 9–33). However, a consensus within the field regarding the composition of the urinary microbiota has not yet been reached. Furthermore, the role of this microbial community in urogenital health and disease, such as urinary tract infection (UTI), is not fully understood.

The UT is the site of the most common bacterial infection experienced in adults. The medical burden of UTI is substantial, with an estimated 1% of all clinical visits in the United States being attributed to UTI management, the cost of which exceeds $3.5 billion annually (34–41). UTI disproportionately affects women, with approximately 50% of women experiencing a UTI in their lifetimes (37, 39). While UTIs are prevalent among young, sexually active populations, the risk of UTI in women also increases with age, leading to an elevated risk in postmenopausal and elderly women. UTIs can be caused by a variety of bacteria and fungi, but the most common pathogens are uropathogenic Escherichia coli (UPEC), followed by Klebsiella pneumoniae, Enterococcus faecalis, and Proteus mirabilis (34). The public’s perception of UTI treatment assumes that it is a simple matter of clearing the infection with antibiotic therapy (36, 42). However, antibiotic resistance and/or allergy is frequent, and UTIs often recur, becoming prolonged cycles of infection, known as recurrent UTI (rUTI), that dramatically reduce quality of life (37, 43). UTIs that ascend to the kidneys can lead to pyelonephritis and life-threatening urosepsis. Some cases of rUTI can last decades and, when refractory to antibiotic therapy, may ultimately require removal of the bladder (34).

Current treatments for UTI and rUTI rely primarily on antibiotic therapy to eliminate the pathogen and achieve UT sterility (34, 43). Despite advances in confirming the existence of the urinary microbiome, conventional antimicrobial strategies for treating UTI and rUTI do not include the preservation or restoration of the microbial community that exists in the host healthy state. This may be because little functional knowledge exists about whether or how the urinary microbiota confers protection against infection. However, the microbial communities resident in many body sites are known to play critical roles in preserving host physiology and health. Disruptions in the resident microbiota are associated with defects in host health, such as inflammatory bowel disease, bacterial vaginosis, cancer, and various metabolic diseases (44–48). The host and resident microbial communities are now believed to be components of a larger composite organism, the holobiont (49, 50). Given such a reciprocal relationship, changes in host physiology or microbial ecology likely affect the system as a whole. This review focuses on the current advances in understanding the microbial and genomic ecology of the human urinary microbiome in the context of how it contributes to host health. A particular focus is placed on the microbiota present in female urine, a microbial community with great potential to inform novel strategies for the diagnosis and therapy of UTI, a disease that affects more than 150 million people per year worldwide (34, 41).

## THE HUMAN BODY IS A COLLECTION OF NICHES WITH UNIQUE MICROBIAL SIGNATURES

The past century has seen significant progress in the functional characterization of the human microbiome. As early as 1908, the concept of a healthy microbiota was proposed by Metchnikoff with his suggestion of enriching the diet with a species of *Lactobacillus* thought to be beneficial in the human gut (46). In 1921, Cannon first characterized compositional changes in the gut flora by diet (46). Today, recent studies have estimated that the female and male human bodies are colonized by 44 × 10^12^ and 38 × 10^12^ individual microbial cells, respectively (51). With the advent of next-generation sequencing (NGS) technologies, significant progress in investigating the human microbiome has been made (52). The deployment of such technologies allows for high-throughput culture-independent characterization of the microbiome with minimal bias (53).

**FIG 1:**
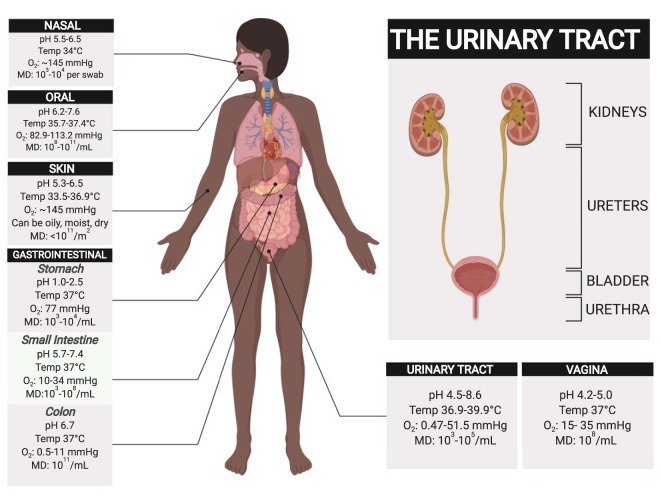
Microbial niches and their environmental characteristics across the human body. Niche conditions for nasal (56–58), oral (51, 59–61), skin (51, 62, 63), gastrointestinal (51, 64, 65), urinary tract (66–69), and vaginal (70, 71) sites are summarized. Abbreviations: Temp, temperature; O_2_, oxygen tension (sea-level average = 760 mm Hg); MD, microbial density (reported in relevant units). This figure was created with Biorender.

The evolution of metagenomics as a tractable and robust tool has fundamentally transformed our ability to characterize the taxonomic and genomic ecology of microbial ecosystems (52). Armed with metagenomics and advanced culturing techniques, numerous research endeavors have improved our understanding of not only which microbes reside within a particular ecosystem but also which genes, metabolic pathways, and physiological adaptations are needed to survive and thrive within a particular ecosystem (1, 2). Rather than searching for core taxa that may be enriched in a particular microbial ecosystem, the research focus is now switching to include the identification of the core gene sets and metabolic pathways needed for a microbial community to function within a niche (54). The genetic signature of a resident microbiota may encode functions that the host cannot perform, giving a competitive advantage to the host. At the time of writing of this review, the Human Microbiome Project (HMP) had profiled the resident microbiota at 18 distinct body sites of more than 250 healthy young individuals using both 16S rRNA gene amplicon sequencing and whole-genome shotgun metagenomics (1–3, 55). Among the accomplishments of the HMP is the first detailed metagenomic characterization of microbial niches throughout the human body. A critical observation made from these data is that each body site sampled seems to have a unique microbial signature and that no microbial taxa were found to be universally present across all body sites and individuals (1). For example, the mouth, gut, vagina, and skin microbial ecologies are each distinct from one another, and these sites harbor microbial populations that have evolved to survive in the unique chemical, metabolic, and immune environment of each niche (1, 51, 56–71) (Fig. 1).

## THE HUMAN URINARY MICROBIOME: AN UNEXPECTED NICHE COMES INTO FOCUS

Given the advances in our current understanding of the human microbiome, it seems reasonable to propose the existence of microbial communities in any body site which encounters the outside world. However, the decades-old dogma of UT sterility, particularly in the bladder, has only now begun to be recognized as disproven (4). Two seminal studies, by Nelson et al. (33) and Siddiqui et al. (5), used metagenomic approaches to demonstrate that the urine of healthy individuals is not sterile and that a UGT microbiome may exist in healthy female and male adults.

### The environmental niche of the urinary tract.

The human UT can be broadly divided into upper and lower compartments. The upper compartment is comprised of the kidneys and ureters, and the lower compartment contains the bladder and urethra (Fig. 1) (72). The luminal surface of the lower urinary tract, bladder, and proximal urethra, called the urothelium, is a transitional epithelium coated by a thin glycosaminoglycan (GAG) layer (72–77). The apical, differentiated cells of the urothelium, called umbrella cells, function to form an impermeable barrier between urinary waste products and underlying body tissues (78). Umbrella cell transmembrane proteins called uroplakins contribute to the barrier function of the urothelium by forming dense plaques on the apical surface of the umbrella cells (73). Because of the sheer force exerted by urinary flow, the ability to bind uroplakins via type 1 fimbriae is a critical virulence factor of UPEC; however, it is unknown if the UT microbiota also utilizes uroplakins as adhesion sites (79, 80). The spatial organization of the UT microbiota has not been assessed.

Factors influencing microbial colonization include environmental characteristics, such as pH, oxygen tension, osmolarity, nutrient availability, adhesion sites, and immune interaction (81). Urine pH varies among individuals and is usually acidic, although healthy urine pH can range from 5 to 8 (82–84). Given that many bacteria possess strictly aerobic or anaerobic metabolism, oxygen availability in the UT may play a role in shaping the ecology and spatial organization of the UT microbiota (69, 85, 86). Arterial blood, for example, exhibits an observed range of oxygen tension of 75 to 100 mm Hg in healthy adults, while the UT oxygen tension ranges from 0.47 to 51.5 mm Hg (Fig. 1) (69, 85, 86). Shannon et al. recently reported that variation in the bladder urinary oxygen tension is correlated with compositional changes in the urinary microbiota (69). Importantly, to support microbial growth, the UT must contain a replenishable nutrient source (83, 87). In a healthy voiding state, the UT is a chemostat, constantly filling and voiding urine. The flux of new urine presumably supplies nutrients to support resident microbes (88). Human urine is composed of many soluble elements, including electrolytes, osmolytes, amino acids, and carbohydrates. To date, a catalogue of more than 2,600 compounds has been detected in urine (83, 87). However, there are other possible sources of nutrients within the UT. The urothelium is covered by a thin layer of GAGs that serves to lubricate and protect the underlying tissue (73–75, 89). The composition of the human UT GAG layer is not fully understood. However, vaginal and gut mucosal epithelia are coated with a layer of excreted, viscous material comprised primarily of amino acids, mucins, various GAGs, and other complex carbohydrates (75, 89–92). Interestingly, many human commensals, such as species of the genera *Lactobacillus*, *Bifidobacterium*, and *Streptococcus*, known to colonize the UT express enzymes that degrade various extracellular GAGs into smaller metabolizable sugars (93–95). The metabolic specializations of UT-resident communities require further mechanistic studies to yield insights that may be clinically translated (83).

### How do we study the human urinary microbiome?

Efforts to study and characterize the human urinary microbiome have resulted in the development of techniques aimed at accurately capturing a microbial community that is challenging to study (Table 1). The human urinary microbiome generally has low biomass in the absence of a UTI, harboring anywhere from <100 to <10^5^ CFU per milliliter of urine (39). Therefore, efforts to profile a resident microbial community must take into account limited starting materials in urine samples, a concept that is well reviewed by Karstens et al. (67). A major consideration for any study assessing the microbiome is the sampling technique used. Three major sampling techniques have been used over the past 10 years to obtain urine samples proposed to represent the urinary microbiota and involve the collection of midstream clean-catch urine (CC) and the collection of urine through a transurethral catheter (TUC) and via suprapubic aspiration (SPA) (Table 1). Each of these methods has advantages and disadvantages. Most sampling techniques introduce perturbations to local microbial communities. With any sampling technique, it is important to consider that sampling order can result in the contamination of sequential sampling sites.

**TABLE 1:** Methods and resources for studying the urinary microbiome^*a*^

Topic	Methods	References^*b*^
Study design	Cross sectional	10, 21, 53, 137–139
	Longitudinal	2, 44, 53, 103, 137, 138
Sample collection	Voided/midstream clean catch	5, 9, 16, 17, 27–32, 67
	Transurethral catheterization	4, 9, 10, 13, 16, 18, 19, 22, 24, 31, 67
	Suprapubic aspiration	9, 67
Data and metadata acquisition	Culture based	4, 13, 19
	16S rRNA amplicon sequencing	4, 5, 9, 10, 13–18, 21, 22, 24, 26–33, 67
	Whole-genome metagenomics	11, 13, 15, 25, 53, 67
Data analysis	Taxonomic profiling	4, 5, 9, 10, 13–18, 21, 22, 24, 26–33, 140
	Functional analysis	11, 13, 15, 25, 53
	Meta-analysis	53, 134, 135, 138, 141, 142
Data democratization	Data curation, structure, public availability and ethics	53, 134, 142–144

a The table presents a summary of selected topics, methods, and references relevant to studying the human UT and UGT microbiome.

b References 53, 67, 134, 137 to 139, and 140 to 144 consist of relevant technical reviews.

For CC, urine is obtained after it has passed through the entire lower urinary tract, and the advantage of the use of CC is that it may be collected by the least invasive means (9, 29). However, this method can suffer from skin, vaginal, and perineal contamination of the urine sample if the periurethral area is not sterilized before urine collection (67). A higher likelihood of contamination lends CC more toward use in the study of the UGT microbiota. Groups studying the spatial organization of the UT-specific microbiota have used a TUC to sample the bladder and urethral microbiota. The use of a TUC has the advantage of more specifically sampling the urinary microbial communities than the use of CC does. However, the insertion of a transurethral catheter to access the bladder disrupts and samples the urethral microbiota (67). One technique to decouple the perturbed urethral microbiota from the bladder-resident microbiota is to take urethral swabs to assess contamination. Finally, suprapubic aspiration (SPA) is the most accurate method to specifically sample the bladder-resident microbiota. However, it is also the most invasive and can be difficult to justify ethically, particularly among elderly populations (6, 9). During SPA, the bladder is accessed via insertion of a needle through the skin of the suprapubic area, just above the pubic bone, and urine is collected directly from the bladder (9). Indeed, Wolfe et al. found in a 2014 study that, when the use of a TUC and SPA was compared, SPA appeared to be the best method to minimize vulvovaginal and urethral contamination of the urine samples for the purposes of studying the bladder microbiome (9). However, the use of a TUC has also been reported to produce results similar to those produced by the use of SPA (6). Balancing the advantages and disadvantages in sampling techniques is a critical step in choosing methodologies for future studies.

### Urine microbial culturing.

Typically, culture-dependent methods are used to test for the presence of uropathogens in urine samples. A critical advantage of urine culturing techniques is the ability to identify and verify viable bacterial populations, which have been done with samples collected with a TUC from the female UT by Hilt et al. (4). The diagnosis of UTI mainly relies on standard urine culture, a method that involves plating urine onto agar plates containing 5% sheep blood agar as well as MacConkey agar plates and incubating aerobically at 35°C for 24 h to obtain quantitative colony counts (4). However, this method has significant limitations in terms of the detection of bacteria in urine, with diagnostic thresholds being ≥10^5^ culturable CFU/ml (96). Studies utilizing both 16S rRNA amplicon sequencing and advanced urine culture indicate the presence of viable bacteria in approximately 90% of samples with no growth by standard urine culture (4) (Table 1). New approaches, like enhanced quantitative urine culture (EQUC), have advanced our understanding of both pathogenic and commensal species within the UT and UGT microbiota (4, 9, 24). This technique includes the use of various combinations of different urine volumes, incubation times, and culture media, as well as aerobic, anaerobic, microaerobic, and CO_2_-enriched atmospheres (19). In contrast, the standard urine culture was designed to detect the major uropathogen E. coli and other bacterial species with similar growth requirements. Direct comparison of these methods in a 2016 study by Price et al. revealed that standard urine culture missed 67% of all uropathogens and 88% of non-E. coli uropathogens detected by the expanded-spectrum EQUC (19). To facilitate the use of EQUC in clinical laboratories, Price et al. developed the streamlined EQUC protocol, which involves plating 100 μl of urine onto blood agar plates, colistin-nalidixic acid agar plates, and MacConkey agar plates in 5% CO_2_ for 48 h (19). The streamlined EQUC protocol was reported to detect 84% of potential uropathogens cultured by the expanded EQUC protocol, whereas standard urine culture detected 33% of potential uropathogens (19). In a 2015 study comparing the urinary microbiomes of women with and without stress urinary incontinence sampled by the use of a TUC, Pearce et al. found that *Lactobacillus*, *Corynebacterium*, *Streptococcus*, *Actinomyces*, and *Staphylococcus* were the most prevalent genera isolated from urine by EQUC, followed by *Aerococcus*, *Gardnerella*, *Bifidobacterium*, and *Actinobaculum* (24). Thomas-White et al. reported that EQUC captured approximately 72% of the genera detected by metagenomics. EQUC did not detect anaerobes from the phyla *Actinobacteria* (*Propionimicrobium*, *Varibaculum*, *Atopobium*), *Firmicutes* (*Peptoniphilus*, *Megasphaera*, *Finegoldia*), or *Bacteroidetes* (*Prevotella*) (13).

### Metagenomic sequencing of the urinary microbiome.

Even EQUC cannot detect the complete spectrum of resident microbial populations within the human UT and UGT. To assess the complete microbial composition without culture bias, researchers have employed NGS-based metagenomic sequencing approaches (3–5, 9, 10, 12, 13, 15, 27, 29, 31). Two major routes of metagenomic sequencing are currently used: 16S rRNA amplicon sequencing and whole-genome shotgun metagenomic sequencing (WGMS) (3, 15, 53) (Table 1). Both techniques rely on NGS technologies. However, 16S rRNA amplicon sequencing uses deep sequencing of amplicons containing the variable regions of the 16S rRNA gene. Downstream bioinformatic analysis allows for sequence identification, community profiling, and ecological structure assessment. An advantage and a disadvantage of 16S rRNA amplicon sequencing are its use of PCR amplification. As a result, 16S rRNA metagenomic sequencing possesses a high sensitivity to detect low-abundance microbial DNA without host contamination (15). However, 16S rRNA amplicon sequencing does not capture genomic sequence information outside the primer-specified amplicon and suffers from intrinsic bias in primer binding, which is not universally efficient across all taxa. Therefore, 16S rRNA amplicon sequencing can reasonably be used only for taxonomic assessment and measurements of relative abundance (15, 67). In contrast, WGMS sequences all the DNA present in a sample. Given a sufficient sequencing depth, WGMS has the potential to comprehensively analyze the whole metagenomic sequence space. WGMS experiments usually suffer from some degree of host contamination (53). For example, Moustafa et al. found that human genomic contamination accounted for 1.3% to 99.9% of the sequencing reads obtained from clinical urine samples (15). It is therefore necessary to sequence samples to a sufficient read depth to account for host contamination and appropriately sample the microbial community (53). Sample preparation as well as DNA extraction techniques can be optimized to enrich for microbial DNA (97, 98). With the whole metagenome available for analysis, it is possible to obtain the community structure and taxonomic profile as well as to functionally characterize the genetic potential of the resident microbiota (53). However, since community taxonomic profiling has been the major goal of most studies of the human urinary microbiome, 16S rRNA sequencing has been the major modality used to date (6).

### The taxonomic profile of the human UT and UGT microbiomes.

Many taxonomic profiling studies have characterized the human urinary microbiome (4, 5, 9–11, 13–19, 21–33). The majority of these studies have used 16S rRNA amplicon sequencing and advanced culture techniques like EQUC to assess taxonomic enrichment between healthy and disease states (4, 5, 9) (Table 1). The major taxa identified in the healthy urinary microbiome are species generally considered to be fastidious, slow-growing microbes (5, 6, 9). Most belong to five major phyla, *Firmicutes*, *Bacteroidetes*, *Actinobacteria*, *Fusobacteria*, and *Proteobacteria* (5), and frequently include the genera *Lactobacillus*, *Corynebacterium*, *Prevotella*, *Staphylococcus*, and *Streptococcus* (6). Many studies have observed a high level of variability between individuals (31). As a result, the members of a core microbial community have not yet been defined.

Several studies using CC or mixed sampling techniques have identified differences between the male and female urinary microbiota (15, 17, 31). For example, Fouts et al. reported that the male urinary microbiota is significantly enriched with the genus *Corynebacterium*, which is typically associated with the skin microbiome. This finding could be related to contamination from skin when CC is used (31). Interestingly, numerous studies have found that the female urinary microbiomes are frequently dominated by the genus *Lactobacillus* (6). Data from a study by Thomas-White et al., using EQUC and whole-genome sequencing of bacterial isolates from urine collected with a TUC, suggest that the vaginal and UT microbiomes are interconnected. While it is possible that contamination during sampling could confound these results, the authors of this study demonstrate that the UT and vaginal microbiotas not only share clonal pathogens but also share commensal organisms associated with vaginal health (13). In healthy women of reproductive age, the vaginal microbiome is usually dominated by species belonging to the genus *Lactobacillus* (99). Lactobacillus crispatus, L. iners, L. gasseri, and L. jensenii are the most prevalent lactobacilli found in the vaginal microbiome (100). The vaginal microbiome is known to play a key role in the maintenance of vaginal pH and the prevention of various urogenital diseases (47, 99, 101). Evidence suggests that vaginal lactobacilli confer a level of protection against bacterial vaginosis, sexually transmitted diseases, and UTIs (47, 99). Continuing research focuses on deciphering the mechanistic basis of protection (47, 100–103).

The temporal stability of the microbial populations within the human UT and UGT remains relatively unknown, as most of the studies performed to date have employed a cross-sectional cohort design (Table 1) (73). Future work is needed to understand how the urinary microbial community changes with both short- and long-term observation intervals. The bulk of studies characterizing the urinary microbiome have focused on urological disorders that disproportionately affect women, such as UTI, urinary incontinence, and pelvic organ prolapse (6, 7, 10, 12, 13, 16, 22, 24). This bias has led to the more thorough characterization of the female urinary microbiome. Although the urinary microbiome has now been taxonomically profiled, the high variability, the lack of a clear association with the host phenotype, and unknown temporal dynamics have left significant areas of opportunity for functional analysis by WGMS and longitudinal observation.

## THE FEMALE URINARY MICROBIOME IN THE CONTEXT OF UTI AND rUTI

UTIs are one of the most commonly diagnosed infections in women and are second only to respiratory tract infections in postmenopausal and elderly women (39). Uncomplicated UTIs are not associated with catheterization or UT abnormality. The incidence of uncomplicated UTI is higher among women than among men across all age groups. Young, sexually active women report incidence rates ranging from 0.5 to 0.7 per person-year, while age-matched men report an incidence rate of about 0.01 per person-year (39). The relationship between the compositional dynamics of the urinary microbiome and the incidence of UTIs is currently an active area of research.

The pathobiology of rUTI is not completely understood; however, the disease can be modeled by an oscillating pattern of relapsed infection interspersed with periods of remission between infections (38). There are two main models to explain the etiology of rUTI: repeated ascending infections from a reservoir outside the UT or reemergence from a persistent population residing within the UT (104). Evidence for both models exists (105, 106). For example, Forde et al. recently showed the dynamics of an E. coli sequence type 131 strain over a 5-year period from an elderly female patient with rUTI and provided evidence of an intestinal reservoir for recurrence (107). On the other hand, Hannan et al. (108) and De Nisco et al. (109) showed the existence of persistent uropathogen populations within the bladder walls of mouse models and human patients, respectively, and a study by Schreiber et al. observed a high incidence of same-strain rUTI in a cohort of 14 women with frequent rUTI (110). Identifying the mechanistic signals that trigger the reemergence of persistent bacterial populations within the UT is critical to understanding the pathogenesis of rUTIs. Interestingly, in a 2017 report, Gilbert and colleagues showed that transient exposures to the vaginal microbiota, particularly Gardnerella vaginalis, can awaken quiescent UPEC reservoirs, leading to reinoculation of the bladder lumen, epithelial cell death and exfoliation, and kidney damage (111).

The relationship between the urinary microbiota compositional dynamics and UTI pathobiology is beginning to be closely studied in humans (15). There is evidence that both the infection itself and treatment strategies, such as antibiotic therapy, affect the urinary microbiome (15, 112, 113). To date, few metagenomic studies have directly profiled the urinary microbiome during UTI; as a result, it remains unclear how the urinary microbial community changes during and after infection. Advanced culturing techniques have been used to characterize the viable urinary microbiota during infection. The current progress in this area has largely been in establishing robust procedures for use in research and clinical laboratories (19). However, Price et al. have reported a differential abundance of several species between patients experiencing UTI and healthy controls (19). For example, K. pneumoniae, Streptococcus agalactiae, Aerococcus urinae, E. faecalis, E. coli, Staphylococcus aureus, and Streptococcus anginosus were found to be enriched in the UTI group compared to the no-UTI group (19). While it is valuable to identify and culture uropathogenic species, characterization of the whole ecology of the urinary microbiome during UTI currently requires the use of NGS-based metagenomics approaches. In 2014, a report on the first use of WGMS of clinical urine samples from 23 patients suspected of having a UTI was published (25). In this study, Hasman et al. detected polymicrobial communities in many of the samples that they analyzed (25). The authors were also able to identify, annotate, and quantify the abundance of antimicrobial resistance genes in these microbial communities (25). In a 2018 study, Moustafa et al. used both 16S rRNA amplicon sequencing and WGMS on 121 CC samples to profile the bacterial, viral, and eukaryotic components of the UGT microbiome (15). Their analysis identified three microbial signatures within patient urine. Two signatures were not associated with UTI and were dominated by *Actinobacteria* and *Firmicutes*. The UTI-associated microbiota signature was dominated by *Proteobacteria* and included many canonical uropathogens, such as UPEC, *Klebsiella*, *Pseudomonas*, and *Enterobacter*. Importantly, the analysis of UTI-associated metagenomes also detected several novel taxa associated with infection, such as *Acidovorax*, *Rhodanobacter*, and *Oligella*. The authors also noted the detection of mammalian viruses and bacteriophage, indicating that further study of the viral component of the urinary microbiome is needed to fully characterize the ecosystem (15). A distinct lack of WGMS analysis exists for urine from rUTI patients. Future, longitudinally designed WGMS studies would greatly aid in the fundamental understanding of the microbial ecology and pathobiology of rUTIs.

Clinical labs often dismiss urine cultures exhibiting multiple colony types, as they consider these results to be from periurethral or vaginal contamination. However, work characterizing polymicrobial infections of the UT has highlighted the potential importance of these previously underappreciated culture results. Polymicrobial UTIs occur more frequently in elderly or immunocompromised populations (114). A polymicrobial ecosystem facilitates the development of synergistic microbial relationships. Interestingly, murine models of polymicrobial UTI have shown that P. mirabilis and Staphylococcus saprophyticus act synergistically during coinfection to increase the incidence of ascending pyelonephritis (115). Similarly, it has been shown that P. mirabilis and UPEC act synergistically in murine coinfection through metabolic interactions that mutually enhance bacterial colonization and persistence (116). In a 2016 report, Keogh et al. showed a remarkable synergistic relationship between UPEC and E. faecalis, also a known gut commensal, in iron-limiting environments (117). E. faecalis secretes a metabolic cue, l-ornithine, that stimulates the biosynthesis of iron uptake machinery by UPEC (117). Taken together, these studies highlight the pathogenic potential of synergistic polymicrobial relationships within the UT and UGT, which may potentially be targeted for new UTI therapies (114).

Current rUTI therapeutic strategies include long-term antibiotic management, topical estrogens, and surgical interventions, like electrofulguration of trigonitis (37, 43, 104, 118). However, long-term or frequent antibiotic therapy remains the frontline treatment strategy (43, 104). Interestingly, Schilling et al. showed in a 2002 report that 10 days of therapy with trimethoprim-sulfamethoxazole, a common first-line antibiotic for the treatment of UTI, was insufficient to eradicate bacteria from bladder reservoirs in a mouse model of UTI (119). Emphasis is now being placed on using appropriate strategies of antimicrobial selection and stewardship for the treatment of UTI and rUTI (120). A legacy of the dogma of UT sterility is the use of antibiotics to treat related but potentially benign conditions, like asymptomatic bacteriuria (ASB), a common clinical observation of a positive urine culture in the absence of symptoms (121). Future whole-genome metagenome studies must shed light on how current paradigms of antibiotic therapy select for antimicrobial resistance and aim to differentiate the metagenomic signatures of UTI and ASB. In a 2019 report, Mulder et al. used CC to assess the effect of antibiotic therapy on the urinary microbiome composition of elderly people and found that antibiotic therapy was associated with an altered composition of the urinary microbiome (112). Interestingly, they noted that the genus *Lactobacillus*, which may be associated with UGT health, was significantly depleted in patients with a recent history of antibiotic use, while uropathogenic species were significantly enriched (112).

Modulation of the UGT microbiome with probiotics has shown promise as a possible route toward novel UTI therapeutics (122). A few studies suggest that the vaginal microbiome may support UGT health by acting as a reservoir for protective commensal species against UTI and rUTI (13, 122). The female UT, UGT, and vaginal microbiotas are frequently enriched in the genus *Lactobacillus* (6, 9, 17, 22). In the vaginal microbial niche, lactobacilli are known to act as a protective microbial population through the secretion of lactic acid, which modulates the local chemical environment (71, 103, 122–125). The antimicrobial and protective properties of *Lactobacillus*-produced lactic acid have been associated with species that secrete large quantities of the d-(−)-isomer, such as L. crispatus (71). Edwards et al. have shown that a vaginal L. crispatus isolate confers protection against Chlamydia trachomatis infection, while Lactobacillus iners, which does not have the ability to synthesize d-(−)-lactic acid, does not confer protection (47). In a 2011 randomized, placebo-controlled clinical study, Stapleton et al. found a moderate reduction in the rUTI incidence in patients given a vaginal L. crispatus probiotic compared to that in patients given a placebo (122). These observations suggest a protective role for L. crispatus and imply a differential host benefit among colonizing *Lactobacillus* species.

### A microbiome-aware future for management and prevention of UTI and rUTI.

With growing knowledge of the role that the urinary microbiota plays in urogenital health, it is becoming clear that UTI and rUTI treatment paradigms may need to consider the state of the urinary microbiome. The traditional goal of achieving UT sterility in the management of UTI and rUTI may destroy beneficial, protective microbial populations as well as pathogens. Without the beneficial microbiota, the UT may be thrown into a dysbiotic, sensitized state at risk for colonization by uropathogens. Probiotics are therefore a logical route for the development of novel therapies. However, a mechanistic understanding of how the urinary microbiota directly influences host health (and vice versa) must be gained before optimal probiotic microbial consortiums may be formulated. Recently, Lev-Sagie et al. reported that vaginal microbiome transplantation from healthy donors may be a viable therapeutic alternative for women with intractable bacterial vaginosis (48). Given that the vaginal and UT microbial niches are predicted to be highly interconnected, vaginal microbiome transplantation may also be an option for the treatment of rUTI (13). Understanding the relationship between urinary microbial populations and UTI risk is a critical step in developing not only therapeutic options but also diagnostic and prognostic tools. The development of molecular ecological signatures of health may allow the accurate identification of dysbiosis and allow at-risk communities to be screened for proactive interventions.

## FUTURE DIRECTIONS IN STUDYING THE HUMAN UT AND UGT MICROBIOME

Gaining translational insights from the study of the human urinary microbiome will require concentrated efforts to fill gaps in our current understanding. To date, the majority of urinary microbiome studies have used cross-sectional cohort designs in order to compare individuals with a disease state to healthy controls. While they are extremely valuable, cross-sectional cohort designs are often confounded by interpersonal variability and are not able to capture temporal variation. Temporal stability is an important feature of a microbial niche and is incorporated into measurements of dysbiosis (44). Recent evidence shows that some, if not much, of the variability seen in other microbial niches can be attributed to temporal variation (103, 126). Employing longitudinal cohorts with participants enrolled to provide urine samples over a rationally defined time course would establish the baseline temporal variation in the urinary microbiota and shed light on the contribution of periodic changes in host physiology, such as menstruation and UTI, on the urinary microbiome (Table 1).

The use of 16S rRNA amplicon sequencing has been the major strategy in profiling the human urinary microbiome for 10 years, yet a core microbiome has not been established for the microbial niches of the bladder, UT, or UGT. Rather than identifying a core taxonomic enrichment, it may be more informative to establish a core genetic enrichment, which requires the use of WGMS. The gut microbiome is, by far, the most-studied human microbial niche (51). In a 2010 report of the MetaHIT project, Qin et al. reported the identification of 3.3 million nonredundant microbial genes from an estimated 1,150 microbial species from human fecal samples using WGMS (54). The catalogue of microbial genes within the human gut microbiome is approximately 150 times the size of the human genome and possesses a diverse range of functional potential. Of particular interest, the authors identified a core, minimal metagenome possessed by the human gut microbiota. Encoded within the gut minimal metagenome are genes and metabolic pathways that complement host physiology, such as the fermentation of sugar to short-chain fatty acids. Similarly, identifying the genetic potential of the urinary microbiome using WGMS will bring insight into which genes or metabolic pathways are required in the UT and UGT to establish host health. Clinical medicine will also benefit from the integration of WGMS, as the genetic assessment of antimicrobial resistance could enrich antimicrobial stewardship practices (25). A current area of research opportunity within the field deals with modeling the community dynamics of UT-resident microbial populations during infection. Commonly used sequencing depths are sometimes not sufficient to profile less abundant, UT microbial populations during UTI. Deeper sequencing of metagenomes and calculation of absolute abundances may help ascertain the fate of putative commensal populations during a UTI (67).

Host-microbe interactions in the UT have been defined by the model systems available to the research community. A critical feature of a model system is that it recapitulates the relevant physiology. To date, the modeling of human UT diseases, such as UTI, has relied primarily on mouse models, in which a TUC is used to inject high-density solutions of a uropathogen into the bladder. These models have been indispensable in defining the mechanisms of bacterial pathogenesis and the host response during UTI (108, 111, 127–132). New model systems, including 3-dimensional cultured organoids and genetically engineered mouse models, that may contribute to the generation of tractable model systems for the mechanistic study of UTI and rUTI are being reported (129, 133). The incorporation of model systems that allow for the study of the immune system in relation to the urinary microbiome will be of great importance to the field. Host immune interactions with the urinary microbiome are currently not well understood and may lead to critical insight into UTI and rUTI pathogenesis as well as maintenance of the urinary microbiome ecological structure.

The public availability of human metagenomic data sets is crucial for translational progress, as it allows for large-scale meta-analyses and independent scrutinization of published results (134). In a 2017 report, Duvallet et al. performed a meta-analysis of 28 published, publicly available gut microbiome data sets from human case-control cohorts (135). They found consistent disease-associated changes in the microbiome composition across multiple independently produced data sets. They also identified common genus-level changes that were shared among multiple disease states, indicating a likely association with nonspecific changes in host health rather than specific clinical associations (135). Data democratization will be critical to the development of consensus microbial community structures of the human UT and UGT in health and disease (Table 1).

As the field moves toward a greater understanding of the urinary microbiome, many patients wait for the advancement that will improve their quality of life. A 2010 study found a significant impact of urinary tract infections on morale and a strong link between UTI incidence and depression among elderly women (136). It is the hopes of many that novel therapies for the management of UTI and rUTI are on the horizon. An understanding of how the urinary microbiome functions in health and disease is critical to this pursuit.
